# Forensic dentistry in human identification: A review of the literature

**DOI:** 10.4317/jced.51387

**Published:** 2014-04-01

**Authors:** Javier Ata-Ali, Fadi Ata-Ali

**Affiliations:** 1 DDS, MS. Public Dental Health Service. Master in Oral Surgery and Medicine. Master in Oral Surgery and Implantology. Valencia University Medical and Dental School; 2 DDS. Valencia University Medical and Dental School

## Abstract

An update is provided of the literature on the role of odontology in human identification, based on a PubMed-Medline search of the last 5 years and using the terms: “forensic dentistry” (n = 464 articles), “forensic odontology” (n = 141 articles) and “forensic dentistry identification” (n = 169 articles). Apart from these initial 774 articles, others considered to be important and which were generated by a manual search and cited as references in review articles were also included. Forensic dentistry requires interdisciplinary knowledge, since the data obtained from the oral cavity can contribute to identify an individual or provide information needed in a legal process. Furthermore, the data obtained from the oral cavity can narrow the search range of an individual and play a key role in the victim identification process following mass disasters or catastrophes. This literature search covering the last 5 years describes the novelties referred to buccodental studies in comparative identification, buccodental evaluation in reconstructive identification, human bites as a method for identifying the aggressor, and the role of DNA in dental identification. The oral cavity is a rich and noninvasive source of DNA, and can be used to solve problems of a social, economic or legal nature.

** Key words:**Forensic identification, DNA, forensic dentistry, rugoscopy, cheiloscopy, saliva.

## Introduction

Forensic dentistry involves the processing, review, evaluation and presentation of dental evidence with the purpose of contributing scientific and objective data in legal processes. Forensic dentists require knowledge encompassing a number of disciplines, since the dental records obtained can identify an individual or afford the information needed by the authorities to establish neglect, fraud or abuse (1). Dental identification can have three different applications (2):

(a) Comparative identification, in which the postmortem dental records are compared with the antemortem records of an individual in order to establish whether both records correspond to the same person.

(b) The obtainment of dental information to narrow the search for an individual when the antemortem records are not available and there are no possible data referred to the identity of the subject.

(c) Identification of victims following mass disasters or catastrophes.

Traditionally, comparisons have been made between postmortem dental records and the antemortem (living) records (presence of dental fillings, endodontic treatments, crowns or bridges, radiological studies to verify the clinical findings, the presence of malocclusions or dental fractures, etc.) to determine whether both records correspond to the same individual. Such techniques are now less widely used, however, due to the increased efficiency and availability of molecular biological techniques (3). In this context, the enamel and dentin layer isolate the pulp cavity from the exterior, thereby affording a valuable source of DNA (4). A number of identification techniques are used by forensic dentists, including rugoscopy, cheiloscopy (lip prints), the obtainment of imprints, or the use of molecular techniques such as polymerase chain reaction (PCR) for analyzing the DNA contained in dental pulp tissue (5).

The present study analyzes the literature published during the last 5 years, offering a description of the novelties referred to buccodental studies in comparative identification, buccodental evaluation in reconstructive identification, human bites as a method for identifying the aggressor, and the role of DNA in dental identification.

## Material and Methods

A PubMed-Medline search was made of the last 5 years (1 October 2007 to 1 October 2012) and using the terms: “forensic dentistry” (n = 464 articles), “forensic odontology” (n = 141 articles) and “forensic dentistry identification” (n = 169 articles). Apart from these initial 774 articles, others considered to be important and which were generated by a manual search and cited as references in review articles were also included. In selecting the studies, we reviewed the titles and abstracts to identify relevant publications, of which the complete text was then obtained. The publications generated by the search were divided into three groups: buccodental studies in comparative identification, buccodental evaluation in reconstructive identification (determination of age; rugoscopy and cheiloscopy; determination of gender), human bites as a method for identifying the aggressor, and the role of DNA in dental identification.

## Buccodental Study in Comparative Identification

Provided the antemortem records are available for comparison, the dental identification process allows us to identify an individual (2). Such records may consist of study models, X-rays or dental treatments such as restorations. Recently, an intelligent dental identification system (IDIS) has been developed that increases the efficacy and shortens the dental identification times with small margins of error (0-1.19%) (6). The similarities and discrepancies between the antemortem and postmortem records must be taken into account in the comparative process. The discrepancies may be either explainable (e.g., a mesio-occlusal silver amalgam filling found to be mesio-occlusal-distal after death) or unexplainable (e.g., the presence of a tooth in the postmortem records that appears as missing in the antemortem records) – in which case identification is discarded (7).  Table 1 shows the different types of identification established by the American Association of Forensic Dentistry (8).

**Table 1:**
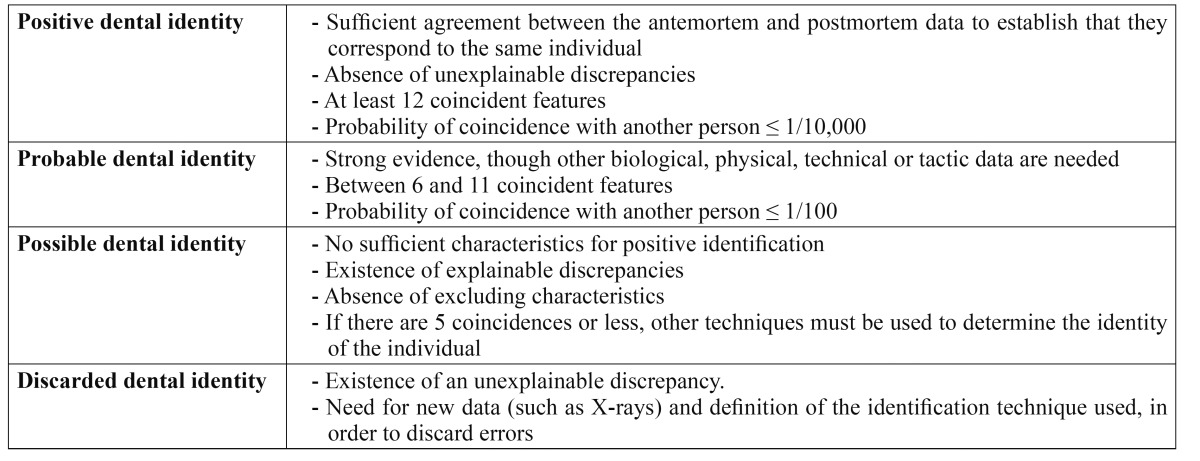
Types of dental identification (8).

The available statistical data indicate that the dental methods contribute to the identification of major catastrophe victims in up to 80% of the cases (9). The percentage of identifications based on dental methods in major catastrophes depends on the nature of the catastrophe, the nationality of the victims, the incidence of the different types of dental treatments, the availability of adequate dental records, and the degree of deterioration of the teeth (9).

## Buccodental Study In Reconstructive Identification

- Determination of age

The teeth with their different development stages offer a noninvasive method for determining the age of an individual (10). In the year 1950, Gustafson (11) was the first to publish a method for estimating the age of a person from the teeth, based on 6 criteria related to hard dental tissue changes that progress with advancing age: occlusal wear, secondary and tertiary dentin layers, cement thickness, the extent of root resorption, the length of the root transparency, and the height of gingival attachment. Gustafson assigned a score of 0-3 to all these factors (according to intensity) - the results being subjective, however, since the scores were not included in an integrating scale. Lamendin *et al*. (12) in turn established a technique for estimating the age of an adult using single-root teeth. This system involved the measurement of two parameters related to age: gingival recession and root transparency (a phenomenon not seen before 20 years of age, and which is due to the formation of hydroxyapatite deposits within the dentinal tubules). These authors measured the maximum length of the transparency on the vestibular surface of the root, which is where the phenomenon is most apparent. The mean error associated with this technique is significantly lower than in the case of the method developed by Gustafson (11) (8.9±2.2 and 14.2±3.4 years, respectively). Another method for estimating dental age is based on the superposition of dental cement layers, whereby the chronological age of the individual is related to the number of deposited cement layers and to the age of eruption of the tooth. Condon *et al*. (13), based on the analysis of 80 teeth corresponding to individuals of known age, established a correlation rate between true age and estimated age of 78%, with standard errors according to dental class of between 4.7 and 9.7 years. Czermak *et al*. (14) facilitated the search for the best location to calculate the cement layers at microscopic level, based on the software-mediated obtainment of images – thereby reducing the human error factor associated with subjectiveness and fatigue.

The method developed by Dermirjian *et al*. (15) involves evaluation of the degree of mineralization of the mandibular teeth, with the designation to each tooth of a value from A to H depending on its degree of development. This in turn is followed by the designation of a score according to the gender of the individual. Lastly, the values of each tooth are added and compared with a conversion table to establish the chronological age of the subject. Mohite *et al*. (16) studied the radiological and histological changes that take place in mandibular bone with the purpose of estimating the dental age of the individual. Radiologically, and taking the mental foramen as reference, the mandibular ramus was seen to increase in length with advancing age – this process being more gradual after 50 years of age – with a decrease in the alveolar process as measured in the craniocaudad direction. Osteoblastic activity was found to decrease with advancing age, with expansion of the Haversian canal system secondary to increased remodeling within the osteons - this giving rise to increased porosity of the cortical bone. The number of concentric laminas per osteon decreases with age, particularly after 50 years of age.

- Rugoscopy and cheiloscopy

Rugoscopy is an identification technique based on the study and analysis of the number, shape, length, direction and merging pattern of the palatal ridges or rugae (rugosities).  Table 2 shows the rugae classification proposed by Lysell (17) and posteriorly modified by Thomas and Kotze (18).

**Table 2:**
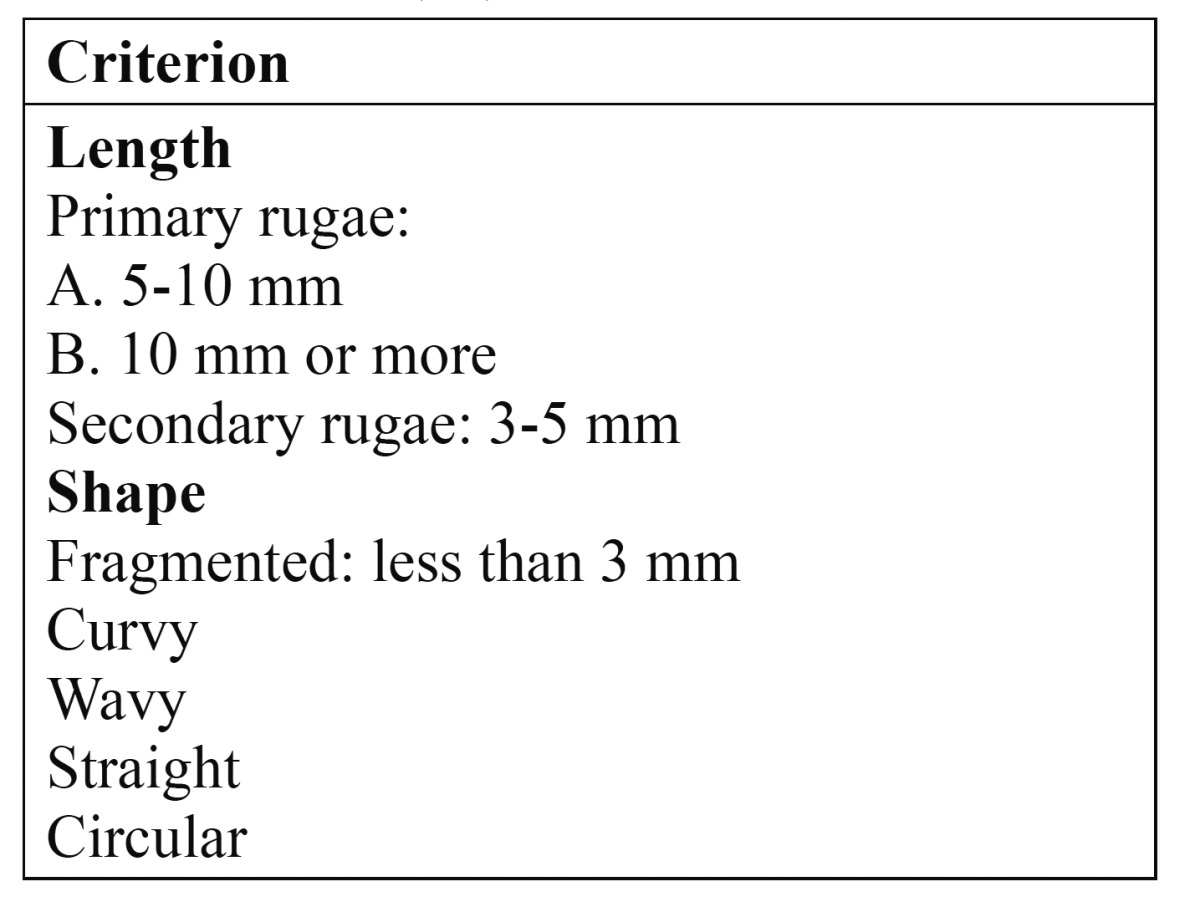
Palatal rugae classification of Thomas and Kotze (18).

The palatal rugae are anatomical ridges, wrinkles or folds located on the anterior portion of the palate, immediately posterior to the upper anterior teeth and the incisive papilla, on either side of the midline (19). The fact that the rugae are present for life, starting from the third month of intrauterine development; are unique to each individual (including twins); and are relatively well protected by the lips, teeth, Bichat’s fat pad and the maxillary bones, implies that they are less affected by decomposition and incineration. As a result, the palatal rugae are studied as a method of identification, comparable to the finger prints of the individual (20). However, a study (21) found that the rugae undergo changes in adolescence, with a marked increase in the number of ridges after 35 years of age. In contrast, another study (17) reported a decrease in the number of rugae after 23 years of age.

According to Ohtani *et al*. (22), three situations complicate identification based on the palatal rugae: changes in rugae height, the presence of flat or poorly accentuated ridges, and the absence of uncomplicated patterns. Nevertheless, other elements can supplement the study of the palatal rugae, such as the incisive papilla, the shape of the mid-palatal raphe, and the palatal tori, where present. One study (22) found the percentage accuracy of identification based on the palatal rugae to be 94%.

**Figure 1:**
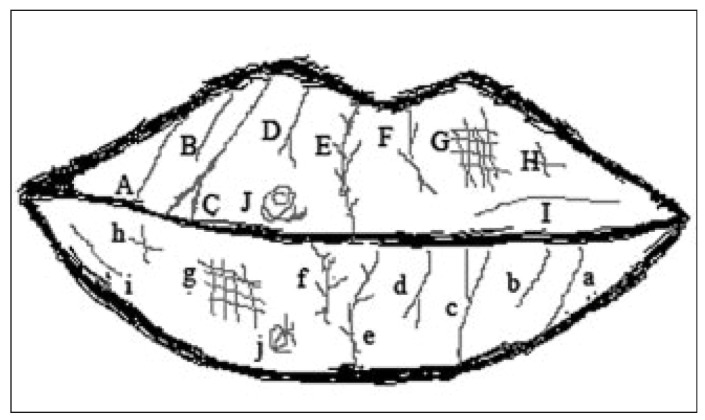
Lip print classification of Renaud (25).

Cheiloscopy involves the study of a series of elevations and depressions that form a characteristic pattern on the lips known as lip prints (23). In the same way as the finger prints, the lip prints are permanent and constant, and are therefore unique to each individual (except monozygous twins) (24). A number of lip print classifications have been developed, such as that published by Renaud (25), which describes 10 types of lip prints (Fig. 1), designated by letters from A to J – capital letters being applied to the upper lip and lowercase letters to the lower lip.

- Determination of gender

The palatal rugae of an individual can be regarded as a complement in the identification of gender. A study (26) based on the methods of Thomas and Kotze (27) and Kapali *et al*. (28) analyzed the number, length, shape and merging pattern of the palatal rugosities, and found convergent rugae to be more common in females and circular ridge morphologies to be more frequent in males. Gender differences were also observed in terms of the number and length of the rugae, though statistical significance was not reached.

**Figure 2:**
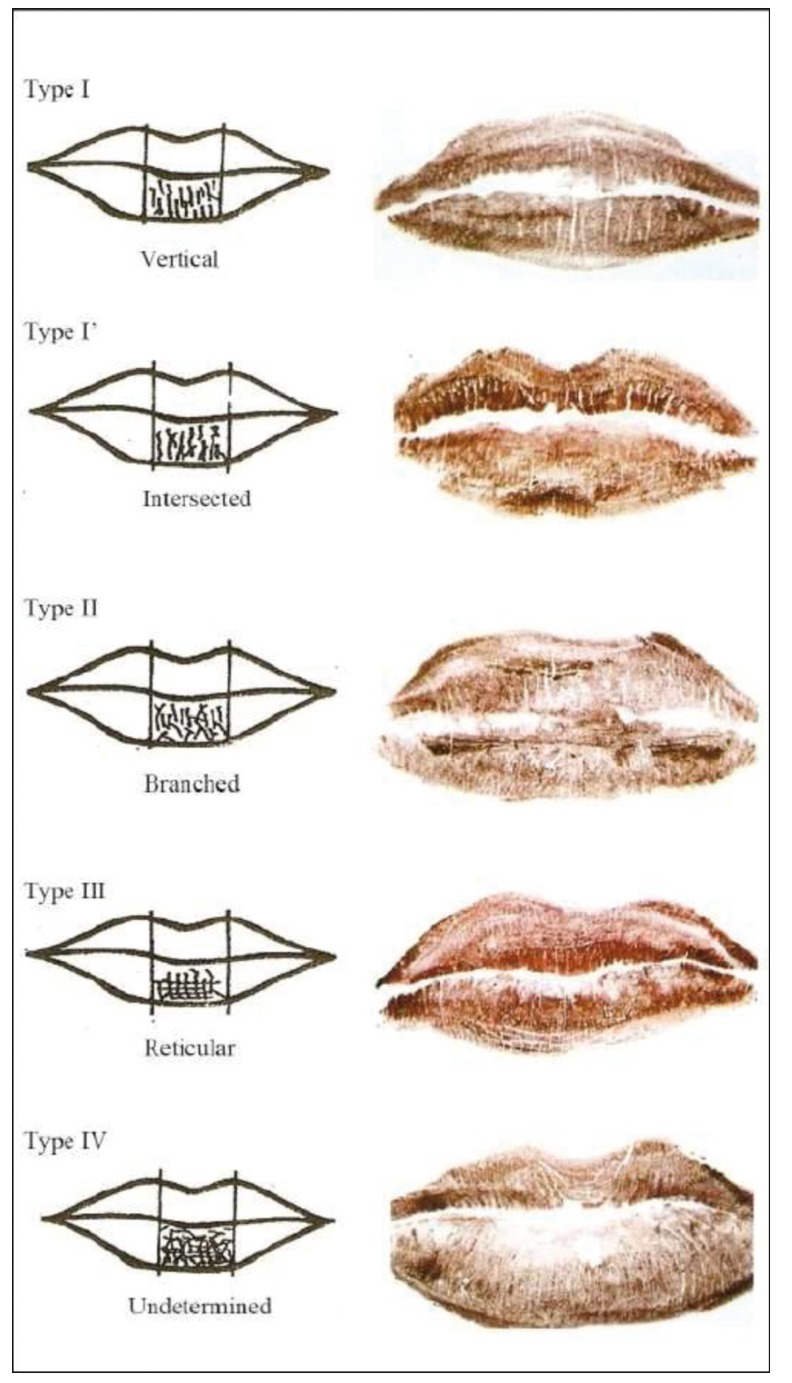
Types of lip print patterns (23).

From a statistical perspective, Archaya *et al*. (29) showed logistic regression analysis to afford a success rate of up to 99.2% in identifying gender on analyzing the shape of the palatal rugae. Sherfydhin *et al*. (30), in a study of canine teeth, recorded statistically significant differences in the lower canines, which were seen to be narrow in females. In turn, the inter-canine distance was shorter than in males. Another study (31) found the size of the crown and of Carabelli’s tubercle to be greater in males. Another alternative for the determination of gender involves the analysis of pulp tissue to establish the presence of chromosome X (32). Lip print morphology can also help in the determination of gender. In this context, females more often present a vertical or intersection-shaped lip print pattern, while ramified or reticular lip print patterns are more frequent in males (Fig. 2). The anatomical differences at skull base level between males and females can also be of help. In this context, the male cranium is significantly larger, thicker and heavier, and of greater capacity than the female cranium, which in turn has softer-contoured and smaller bone crests and protuberances (33). In a study (33) of 100 skull bases (50 males and 50 females), measurements were made of the distances (in mm) between the incisor foramen (IF) and the right greater palatal foramen (RGPF)(IF-RGPF), the incisor foramen and the left greater palatal foramen (IF-LGPF), the right and left greater palatal foramen (RGPF-LGPF), the basion and the incisor foramen (Ba-IF), and the incisor foramen and a middle point between the two major palatal foramens (IF-RGPF/LGPF). Statistically significant results were obtained for IF-RGPF (*p* = 0.020), IF-LGPF (*p* = 0.008), Ba-IF (*p* = 0.004) and IF-RGPF/LGPF (*p* = 0.015), while the findings for RGPF-LGPF failed to reach statistical significance.

## Human Bites as a Method for Identifying an Aggressor

Consensus is currently lacking among forensic dentists as to whether the dentition or behavior of the human skin in response to biting action is characteristic, individual and unique. Nevertheless, many studies have been made to determine whether each human dentition is unique or not (4).

Human bite marks can be found on practically any part of the body (4). While in females human bites are more commonly found on the breasts and legs secondary to sexual assault, in males bite marks are mainly found on the arms and shoulders (34,35). The diameter of the human bite typically varies between 25-40 mm. A central contusion zone is normally observed within the teeth marks. Extravascular bleeding is caused by tooth pressure upon the tissues directed towards the interior from the periphery of the bite mark (4).

The individual bite characteristics must be documented in order to positively identify the suspect. Certain important dental features can include fractures, dental rotations, attrition and wear, congenital malformations, etc. (4). The physical and biological findings deteriorate from the moment of the actual bite, and therefore should be documented as quickly as possible. Saliva is deposited in the skin at the time of biting and should be collected - preferably using the double cotton swab technique (36). Dry saliva is hard to detect, and the amylase test is needed to identify its presence (37).

An exact and precise impression should be obtained of the bite surface to register all the irregularities produced by the teeth upon the skin, employing vinyl polysiloxane, polyether or other impression materials recommended for the obtainment of imprints for fixed prostheses (4).

## The Role of DNA in Dental Identification

The oral cavity is a useful source of DNA. The latter is obtained from saliva, the oral mucosal cells and the teeth. The main DNA source is blood, though in some situations this type of sample is not available for analysis. In teeth, DNA is found in the pulp tissue, dentin, cement, periodontal ligament and alveolar bone (37). Due to the resistance of the hard tissues of the teeth to environmental actions such as incineration, immersion, trauma or decomposition, pulp tissue is an excellent source of DNA (5).

Pulp tissue is the most widely used option, since it is normally abundant and is less vulnerable to contamination by non-human DNA. The pulp tissue samples are collected in three ways: crushing, horizontal or vertical tooth sectioning, and through an endodontic access. Sweet and Hildebrand (38) were pioneers in the obtainment of DNA by tooth crushing through cryogenization.

Pulp tissue is easier to prepare and analyze than other sources. However, in many case the analyzed tooth lacks pulp tissue or may have been endodontically obturated. It also may be contaminated by microorganisms or by non-human DNA. In such cases dentin or cement is used for DNA extraction (37). Forensic dentists should incorporate these new technologies, since a number of methods are available for the extraction of DNA from biological samples, though no standardized protocols for their use have been established to date (39).

## Conclusions

An analysis has been made of the literature published during the last 5 years, offering a description of the novelties referred to buccodental studies in comparative identification, reconstructive identification (determination of age, rugoscopy and cheiloscopy, determination of gender), human bites as a method for identifying the aggressor, and the role of DNA in dental identification. The oral cavity is a rich and noninvasive source of DNA, and can be used for the identification of individuals and for providing information needed in legal processes.
